# Hydrogen sulfide promotes autophagy of hepatocellular carcinoma cells through the PI3K/Akt/mTOR signaling pathway

**DOI:** 10.1038/cddis.2017.18

**Published:** 2017-03-23

**Authors:** Shanshan S Wang, Yuhan H Chen, Ning Chen, Lijun J Wang, Dexi X Chen, Honglei L Weng, Steven Dooley, Huiguo G Ding

**Affiliations:** 1 Department of Gastrointestinal and Hepatology, Beijing You' An Hospital Affiliated to Capital Medical University, Beijing, 100069, China; 2 Cell Biology Laboratory, Beijing Institute of Hepatology, Beijing, 100069, China; 3 Department of Infections Disease, Henan Provincial People's Hospital, Zhengzhou, 450003, China; 4 Department of Gastroenterology, Pinggu Hospital, Pinggu District, Beijing 101200,China; 5 Molecular Hepatology, University of Heidelberg, University Medical Center Mannheim, Mannheim 68167, Germany

## Abstract

Hydrogen sulfide (H_2_S), in its gaseous form, plays an important role in tumor carcinogenesis. This study investigated the effects of H_2_S on the cell biological functions of hepatocellular carcinoma (HCC). HCC cell lines, HepG2 and HLE, were treated with NaHS, a donor of H_2_S, and rapamycin, a classic autophagy inducer, for different lengths of time. Western blotting, immunofluorescence, transmission electron microscopy (TEM), scratch assay, CCK-8 and flow cytometric analysis were carried out to examine the effects of H_2_S on HCC autophagy, cell behavior and PI3K/Akt/mTOR signaling. Treatment with NaHS upregulated expression of LC3-II and Atg5, two autophagy-related proteins, in HepG2 and HLE cells. TEM revealed increased numbers of intracellular double-membrane vesicles in those cells treated with NaHS. Like rapamycin, NaHS also significantly inhibited expression of p-PI3K, p-Akt and mTOR proteins in HCC cells. Interestingly, the expression of LC3-II was further increased when the cells were treated with NaHS together with rapamycin. In addition, NaHS inhibited HCC cell migration, proliferation and cell division. These findings show that H_2_S can induce HCC cell apoptosis. The biological function of the gasotransmitter H_2_S in HCC cells was enhanced by the addition of rapamycin. Hydrogen sulfide influences multiple biological functions of HCC cells through inhibiting the PI3K/Akt/mTOR signaling pathway.

Hydrogen sulfide (H_2_S) is the most recently discovered gas that is synthesized in mammalian cells. Like nitric oxide (NO) and carbon monoxide (CO), which contribute to tissue damage and inflammation,^1, 2^ H_2_S exerts multiple physiological and pathological effects on cell growth, differentiation and proliferation. This gas has been reported to influence cardiovascular remodeling, change vascular active substances, damage the gastric mucosal and have important biphasic effects in cancer.^3, 4^

The liver is one of the most important organs that produce and clear H_2_S.^5^ Endogenous H_2_S participates in the pathogenesis of many liver diseases, affecting processes such as deregulation of hepatic lipid^6^ and glucose metabolism,^7^ oxidative stress,^8^ mitochondrial bioenergetics,^9^ fibrosis,^10^ cirrhosis,^11^ hepatoprotection and hepatotoxicity.^12^ In addition, endogenous or exogenous H_2_S may play an important role in the occurrence and development of liver tumors. In the liver, synthesis and clearance of H_2_S are mainly managed by hepatic stellate cells (HSC), the major cell source of extracellular matrix in liver fibrosis and HCC.^13, 14^

Autophagy is the natural, destructive cellular mechanism that degrades damaged proteins and cytoplasm components in lysosomes and thus maintains cellular homeostasis and supplies substrates for energy generation. It is a critical pathway for homeostasis, development and other pathophysiological processes.^15^ Autophagy plays an important role in the healthy and diseased liver.^16, 17^ Some molecular mechanisms of autophagy have been investigated in liver disease, especially in HCC. For example, the PI3K/Akt/mTOR signaling pathway is reported to play an important role in cell autophagy.^18^ Previous experiments indicated that autophagy is a double-edged sword, acting both to promote and inhibit tumor growth, and development in different experimental settings.

This study investigated the role and underlying mechanisms of H_2_S on autophagy of HCC cells.

## Results

**Figure 1:**
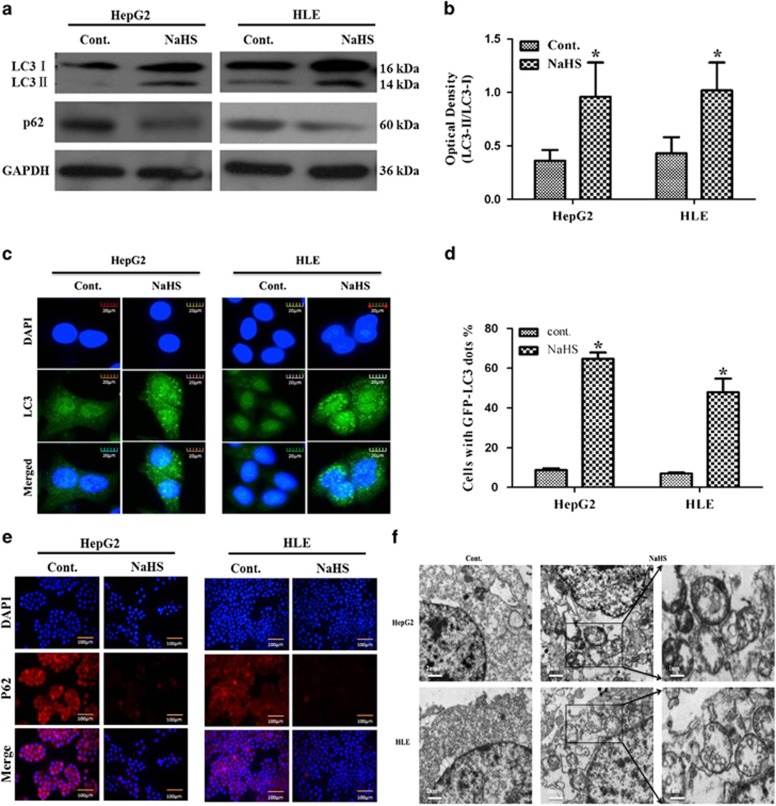
Hydrogen sulfide promotes autophagy of HepG2 and HLE cells. (**a**) Western blotting detected increased expression of LC3-II, Atg5 and P62 autophagy-related protein in the presence of 10^−3^ M NaHS in HepG2 and HLE cells. *β*-actin was used as internal control. (**b**) Densitometric analysis of LC3-II/LC3-I is shown in the histogram. (**c**) HepG2 and HLE cells transfected with GFP-LC3 plasmid after 24 h, LC3 puncta dots (green) were observed under a fluorescence microscope; the nuclei (blue) were stained with DAPI. (Scale bar: 20 *μ*m) (**d**) The percentage of cells presenting typical LC3 puncta dots. (**e**) Expression of P62 as analyzed by fluorescence microscopy in HepG2 and HLE cells treated with NaHS for 24 h (Scale bar: 100 *μ*m). (**f**) Intracellular double-membrane vesicles (arrows), the ultrastructural feature of autophagy, were observed by TEM (Scale bars 2 and 1 *μ*m). Each figure is representative of an experiment that was repeated at least three times. The data represent the mean±S.D. of three samples. *P<0.05 compared with control

HepG2 cells were treated under 10^−4^ M and 10^−3^ M NaHS for 24 h, then western blotting and immunofluorescence staining show that the more significantly effective NaHS dosage was 10^−3^ M (see theSupplementary Figure 1).To examine the effect of H_2_S on HCC cells, HepG2 and HLE cells were treated with NaHS at a final concentration of 10^−3^ M for 24 h, then western blotting, immunofluorescence staining and TEM assays were used to detect markers of autophagy and morphological alterations in these cells. Western blotting demonstrated that H_2_S treatment significantly upregulated protein expression of LC3-I, LC3-II and autophagy-related protein Atg5, and reduced p62 expression in both the cell lines (Figures 1a and b). Immunofluorescence analyses further confirmed that H_2_S drastically increased the expression of LC3 in both HepG2 and HLE cells (Figure 1c). Compared with untreated cells, the percentage of LC3-positive cells increased in HepG2 (64.7±3.15% *versus* 8.77±0.89%) and HLE (47.87±6.93% *versus* 7.0±0.52%) cells after NaHS treatment (Figure 1d, *P*<0.05). Consistent with western blot findings, immunofluorescence staining also demonstrated reduced p62 in 24 h H_2_S-treated cells (Figure 1e). Furthermore, TEM showed increased numbers of mitochondria encircled by double-membrane structures resembling autophagosomes in H_2_S-treated cells than in control cells(Figure 1f).

### H_2_S inhibits cell migration and proliferation, but induces apoptosis

**Figure 2:**
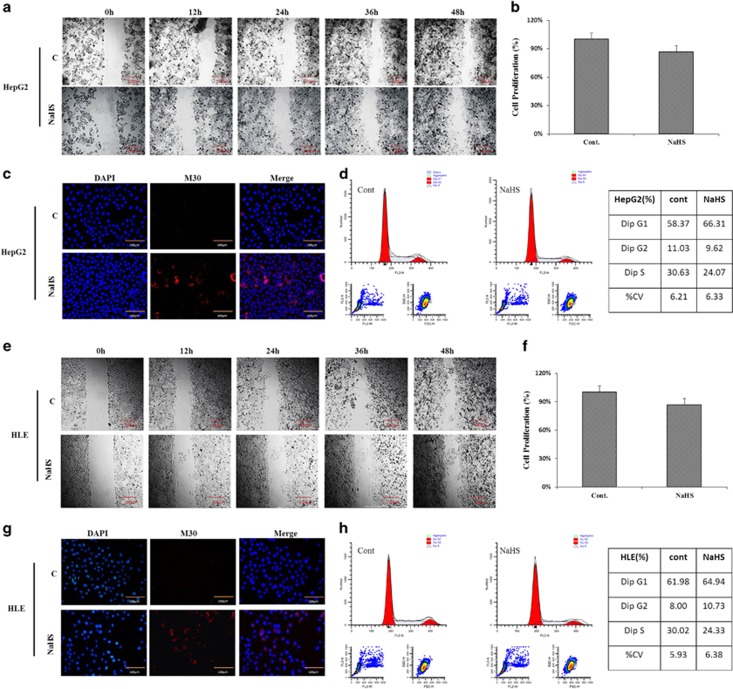
Hydrogen sulfide inhibits cell migration, proliferation and cell cycle progression, but accelerates apoptosis. Scratch assay was used to evaluate cell migration. HepG2 (**a**) and HLE (**e**) cells were treated with NaHS for 48 h then viewed, and images were captured under a light microscope at 0, 12, 24, 36 and 48 h separately (Scale bar: 20 *μ*m). CCK-8 assay was used to verify HepG2 (**b**) and HLE (**f**) cell proliferation. Immunofluorescence was used to evaluate M30 immunoreactivity in HepG2 (**c**) and HLE (**g**) cells. M30 staining was red and nuclei were blue (Scale bar: 100 *μ*m). Flow cytometry was used to analyze the cell cycle of HepG2 (**d**) and HLE (**h**) cells after NaHS treatment for 24 h. The data represents the mean±S.D. of three samples. All the data are representative of an experiment that was repeated at least three times

The effects of H_2_S on HCC cell migration were evaluated by starch test. The results showed that migration of both HepG2 (Figure 2a) and HLE (Figure 2e) cells was slightly inhibited following NaHS treatment for 12 h compared with the control group. The CCK-8 assay showed that proliferation of both HepG2 and HLE cells was also inhibited after 24 h H_2_S treatment (Figures 2b and f). Immunofluorescence staining forM30 protein, an apoptosis-related protein, showed that H_2_S induced early apoptosis in both HepG2 and HLE cells (Figures 2c and g). Flow cytometric analyses further found that when cells were exposed to H_2_S, there were fewer cells in S phase (HepG2: 24.09%±0.37; HLE:24.41±0.19) compared to those without treatment(HepG2: 30.49%±0.15; HLE: 29.97±0.21) (Figures 2d and h).

### H_2_S induces cell autophagy by inhibiting the PI3K/AKT/mTOR signaling pathway

**Figure 3:**
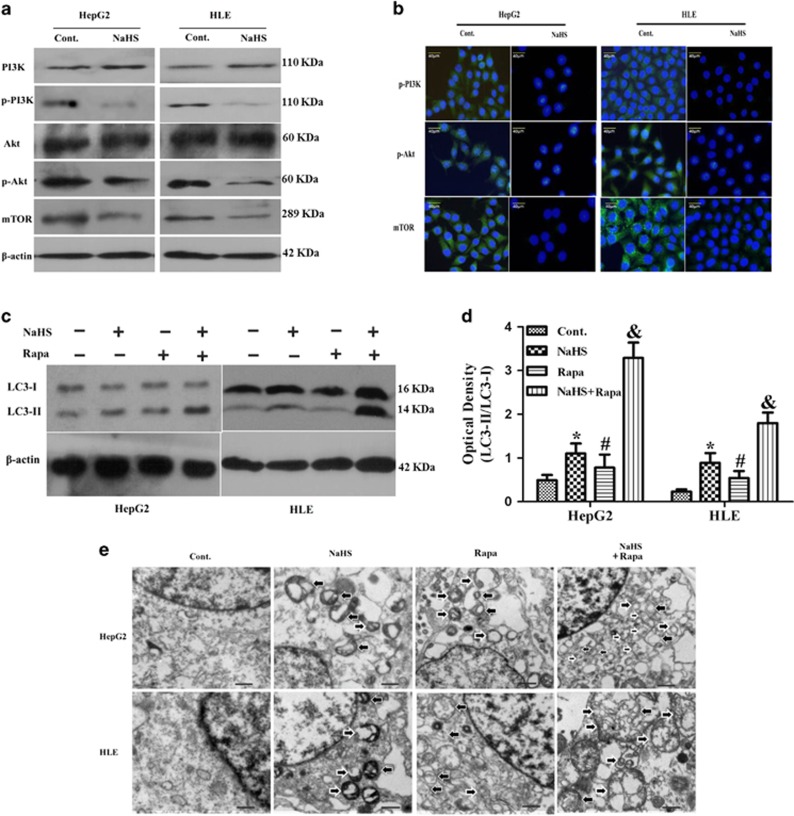
H_2_S promotes cell autophagy by inhibiting the PI3K/AKT/mTOR signaling pathway. (**a**) Western blotting was used to detect the expression of PI3K/ p-PI3K, Akt/p-Akt and mTOR in HepG2 and HLE cells. (**b**) Immunofluorescence staining revealed that p-PI3K/p-Akt/mTOR decreased after NaHS treatment, with the green indicating positive staining for p-PI3K/p-Akt/mTOR (Scale bar: 40 *μ*m). (**c**) Western blotting was performed to detect the expression of LC3-II in HepG2 and HLE cells treated with NaHS alone, rapamycin alone or NaHS in combination with rapamycin. *β*-actin was used as internal control. (**d**) Densitometric analysis of LC3-II was shown as a histogram. The data represent the mean±S.D. of three samples.*P<0.05 compared with control cells; ^#^*P*<0.05 *versus* cells treated with NaHS alone; ^&^*P*<0.05 *versus* cells treated with rapamycin alone. (**e**) Transmission electron microscope observation of cell autophagosomes (Scale bar: 2 *μ*m)

Next, western blotting analyses showed that H_2_S treatment significantly decreased expression of phosphorylated-PI3K (p-PI3K), phosphorylated-Akt (p-Akt) and mTOR (Figures 3a and b) in HepG2 and HLE cells. Given that rapamycin induces cell autophagy by inhibiting mTOR expression, we speculate that like rapamycin, H_2_S induces HCC cell autophagy by inhibiting the PI3K/AKT/mTOR signaling pathway. As expected, the expression of LC3-II was increased by treatment with either H_2_S or rapamycin alone in both HepG2and HLE cells. Combined administration of H_2_S together with rapamycin further significantly increased the expression of LC3-II (Figures 3c and d), probably because both of them influence the PI3K/AKT/mTOR signaling pathway. Consistent with findings from western blotting, TEM further demonstrated that the quantity of double-membrane autophagosomes significantly increased in HepG2 and HLE cells treated with NaHS together with rapamycin when compared to those treated with either compound alone (Figure 3e).

### H_2_S and rapamycin additively inhibit the migration, proliferation and cell cycle of HCC cells

**Figure 4:**
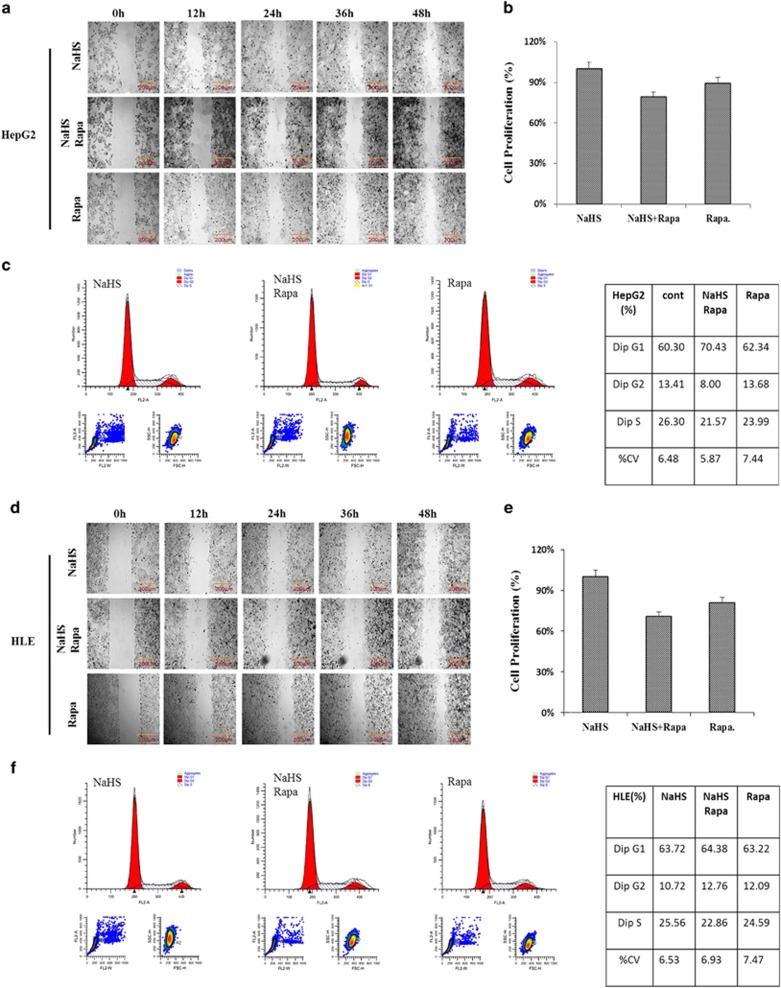
Rapamycin and H_2_S additively inhibit the migration, proliferation and cell cycle of liver cancer cells. Scratch assay was performed to detect HepG2 (**a**) and HLE (**d**) cell migration under different treatments (Scale bar: 200 *μ*m). HepG2 (**b**) and HLE (**e**) cell proliferation was calculated by CCK-8 assay. The data represents the mean±S.D. of three samples. HepG2 (**c**) and HLE (**f**) cells were incubated under different conditions for 24 h then analyzed by flow cytometry to investigate the cell cycle. The data are representative of an experiment that was repeated at least three times

To further test the additive effects of H_2_S and rapamycin on cell migration, proliferation and division, the scratch assay, CCK-8 assay and flow cytometric analyses were carried out. Compared to the individual treatments, combined H_2_S and rapamycin administration further reduced cell migration (Figures 4a and d). CCK-8 assay showed that proliferation of both HepG2 and HLE cells was further inhibited by combined H_2_S and rapamycin treatment (70 and 75%) compared to treatment with H_2_S or rapamycin separately (Figures 4b and e). Both HepG2 and HLE cells underwent obvious cell cycle arrest with combined H_2_S and rapamycin treatment (Figures 4c and f).

## Discussion

The data gathered in this study suggest that treatment of HepG2 and HLE cells with NaHS promoted cell autophagy and apoptosis, and also inhibited cell migration, proliferation and cell cycle progression. Further experimental results suggested that the effects of NaHS may be mediated by the PI3K-Akt signaling pathway, and this was confirmed by the additional effect of rapamycin.

There are two forms of H_2_S in the body: most H_2_S exists as sodium hydrosulfide (NaHS) with a minor amount in the form of the gas.^19^ In this study, we used exogenous NaHS as a donor of H_2_S gas. There has been some previous research carried out into the relationship of H_2_S and autophagy. Gotor *et al.*^20^ reported that in plant cells, sulfide plays a biological role through negative regulation of autophagy, which is unrelated to nutrient deficiency. Another study produced contradictory results, showing that when the H_2_S level increased in chickens, autophagy also correspondingly increased.^21^ In this study, our first result demonstrated that autophagy of HCC cells is enhanced following treatment with 10^−3^ M NaHS for 24 h.

The biological function of H_2_S also has generated a great deal of controversy, with some studies indicating that H_2_S promoted cell proliferation, while others showed that H_2_S induced cell apoptosis. One study found that cell proliferation was stimulated by treatment with low concentrations of H_2_S, but inhibited at high concentrations.^22^ Meanwhile, research in colon cells verified that H_2_S could inhibit cell proliferation and arrest the cell cycle, findings which are similar to our results.^23^ In our second result, we demonstrate that H_2_S treatment of HepG2 and HLE cells can induce apoptosis, inhibit the cell cycle and proliferation, and block cell migration.

One recent study in esophageal cells verified that exogenous H_2_S promoted proliferation, inhibited apoptosis and increased migration through the HSP90 pathway.^24^ Meanwhile, other reports verified that the effects of H_2_S involve the PI3K/Akt signaling pathway in neuroblastoma and cardiac cells.^25, 26^ There are several signaling pathways involved in cell autophagy, such as the PI3K/Akt/mTOR, Bcl2/beclin1, MAPK/Erk1/2, and AMPK signaling pathways. But the PI3K/Akt/mTOR signaling pathway is becoming increasingly difficult to ignore in the investigation into the mechanism of cell autophagy.^27, 28^ In our study, we found that the PI3K/Akt/mTOR signaling pathway is inhibited under NaHS treatment. Rapamycin, which acts through specifically inhibiting mTOR in the PI3K/Akt pathway, is recognized to be a drug which promotes autophagy. Both NaHS and rapamycin reduce the expression of mTOR, so when the two are combined, any increase in autophagy should be more significant. As we envisaged, when rapamycin was added together with H_2_S treatment, the enhancement of autophagy was more significant. These results indicate that the induction of autophagy by H_2_S may be mediated by inhibition of the PI3K/Akt/mTOR signaling pathway. Further functional experimental results confirmed our hypothesis.

The emerging role of H_2_S in liver cancer may open a new therapeutic approach. The toxic effects of H_2_S are well recognized, and recently many studies have added to the evidence that H_2_S may act as a mediator of some aspects of liver function. However, the exact function of H_2_S remains controversial, and its precise mechanism remains to be elucidated. In conclusion, our results demonstrate that H_2_S is involved in liver cancer and that its effects are mediated through thePI3K/Akt/mTOR signaling pathway.

## Materials and Methods

### Cells

HepG2 and HLE cells, two HCC cell lines, were obtained from the Beijing Institute of Hepatology. Cells were cultured in DMEM supplemented with 10% fetal calf serum (FCS) at 37 °C in a 5% CO_2_ incubator.

### Western blotting

Western blotting was performed to analyze the expression of autophagy-associated proteins in HepG2 and HLE cells. Briefly, cells were collected and lysed in ice-cold RIPA lysis buffer (1 × Tris-buffered saline, 1% Nonidet P-40, 0.5% sodium deoxycholate, and 0.1% sodium dodecyl sulfate (SDS)), then 40 *μ*g of cell lysate from each sample was separated by 10% SDS-polyacrylamide gel electrophoresis (PAGE). Electrophoretic transfer of proteins from gels onto nitrocellulose membranes was carried out in a transblotting chamber. Membranes were blocked by immersing in 5% nonfat milk (w/v) in phosphate-buffered saline (PBS) for 1 h to inhibit nonspecific binding before being incubated with primary antibodies at room temperature for 2 h. After rinsing with PBS/0.1% Tween-20, membranes were incubated with horseradish peroxidase-conjugated secondary antibodies (Zhongshan Boil Tech Co, Beijing, China). Immuno-complexes were visualized by incubation using an enhanced chemiluminescence system (Thermo Fisher Scientific, OL191210A, MA, USA) and were exposed on X-ray film. Western blotting was repeated at least three times for each experiment.

### Transient transfection of a GFP-LC3-expressing construct

A GFP-LC3-expressing construct was provided by the Beijing Institute of Hepatology. The GFP-LC3 plasmid was transfected into the cells using the FuGene Transfection Method according to the protocol provided by Roche Diagnostics. The number of GFP-LC3-positive cells was counted manually in five randomly-selected fields under a fluorescence microscope (LEICA CTR5000, Leica Microsystems Ltd, Wetzlar, Germany). Cell nuclei were visualized by staining with 4,6-diamidino-2-phenylindole (DAPI).

### Immunofluorescence

HepG2 and HLE cells were treated with NaHS (10^−3^ M), a donor of H_2_S, for 24 h. Then cells were fixed in ice-cold 4% paraformaldehyde/PBS for 15 min and soaked in 0.5% Triton X-100/ PBS for 20 min. After washing three times with PBS and blocking with 3% bovine serum albumin (BSA) in PBS for 30 min, the cells were incubated with the primary antibodies (anti-P62 and anti-M30) overnight at 4 °C. The next day, the cells were washed in PBS then incubated with the appropriate secondary antibody conjugated with fluorescein isothiocyanate (FITC) or rhodamine (TRITC) for 1 h at 37 °C. Cell nuclei were counterstained with DAPI. Cells were viewed under a fluorescence microscope (Nikon Eclipse 80i; Nikon, Tokyo, Japan). Quantitative analysis of apoptosis was performed by counting more than 500 cells in each sample.

### Transmission electron microscopy (TEM)

Cells were fixed in 2.5% glutaraldehyde and 0.1 M cacodylate buffer (pH 7.4) for 2 h following trypsinization and rinsed twice with precooled PBS. After washing with buffer solution, cells were post-fixed in 1% osmium tetroxide (OsO4) and 0.1 M cacodylate buffer (pH 7.4). Then, the fixed cells were washed with buffer solution, dehydrated through different concentrations of ethanol, and embedded in epoxy resin. The ultrastructures of cells undergoing autophagy were observed and imaged under TEM (JEM-1200; Jeol Ltd, Tokyo, Japan) at 80 kV.

### Scratch test

Suspensions of HepG 2 and HLE cells were pipetted into 6-well microplates at 5 × 10^5^ cells per well. After allowing them to completely attach, the cells were subjected to different treatments as: control, 10^−3^ M NaHS, 10 nM rapamycin and 10^−3^ M NaHS+10 nM rapamycin. Then, the cells were viewed under a light microscope at 0, 12, 24, 36 and 48 h following treatment.

### CCK-8

The Cell Counting Kit (CCK)-8 was used to assess the effect of NaHS and rapamycin on cell proliferation according to the standard protocol from Dojindo Laboratories (Kumamoto, Japan). Briefly, 5 × 10^3^ HepG 2 or HLE cells were pipetted into wells of a 96-well microplate and subjected to various treatments (NaHS and rapamycin) before analysis by the CCK-8 assay. The absorbances were measured at a wavelength of 450 nm on a Universal Microplate Reader (EL x800; Bio-Tek Instruments Inc., Winooski, VT, USA). Cell proliferation was calculated using the formula: Cell proliferation%=((A570 or 450 sample−background)/(A570 or 450 control–background)) × 100%.

### Cell cycle analyses

Before treatment, HepG2 and HLE cells were cultured in serum-free medium for 12 h to arrest the cell cycle, then the serum-free supernatant was replaced by fresh medium containing 10% FCS. The cells were transferred into 12-well plates, treated with NaHS and rapamycin for 24 h, then stained with propidium iodide (PI) at a final concentration of 50 mg/l. DNA content was analyzed with a FACScan-420 flow cytometer (BD Biosciences, Franklin Lakes, NJ, USA). The distribution of cells in different cell cycle stages was determined according to the DNA content.

### Statistical analyses

Quantitative variables are expressed as mean±S.D. on the basis of at least three separate experiments. Statistical significance was calculated by Student's *t*-test (SPSS19.0, SPSS Inc. Chicago, IL, USA). *P*<0.05 was considered significant.
