# Supplementary material for: Hydrogen sulfide promotes autophagy of hepatocellular carcinoma cells through the PI3K/Akt/mTOR signaling pathway
Source: Cell Death Dis. 2017 Mar 23;8(3):e2688–. doi: 10.1038/cddis.2017.18 (PMC5386547; doi:10.1038/cddis.2017.18)
Supplement: Supplementary Figure 1 [file cddis201718x1.docx]

**Legends**

**Supplementary Figure. Different concentrations of Hydrogen sulfide promote HepG2 cells autophagy.**

(**A**) Western blotting detected the increased expression of LC3-II protein in the presence of 10^-4^M and 10^-3^M NaHS in HepG2 cells. GAPDH was used as internal control. (**B**) Expression of P62 as analyzed with fluorescence microscopy in HepG2 cells which treated with 10-4M and 10-3M NaHS for 24h(Size bar: 100μm). \All the figure is representative of an experiment that was repeated at least three times.
