# Supplementary figures and images for: Hydrogen sulfide promotes autophagy of hepatocellular carcinoma cells through the PI3K/Akt/mTOR signaling pathway
Source: Cell Death Dis. 2017 Mar 23;8(3):e2688–. doi: 10.1038/cddis.2017.18 (PMC5386547; doi:10.1038/cddis.2017.18)

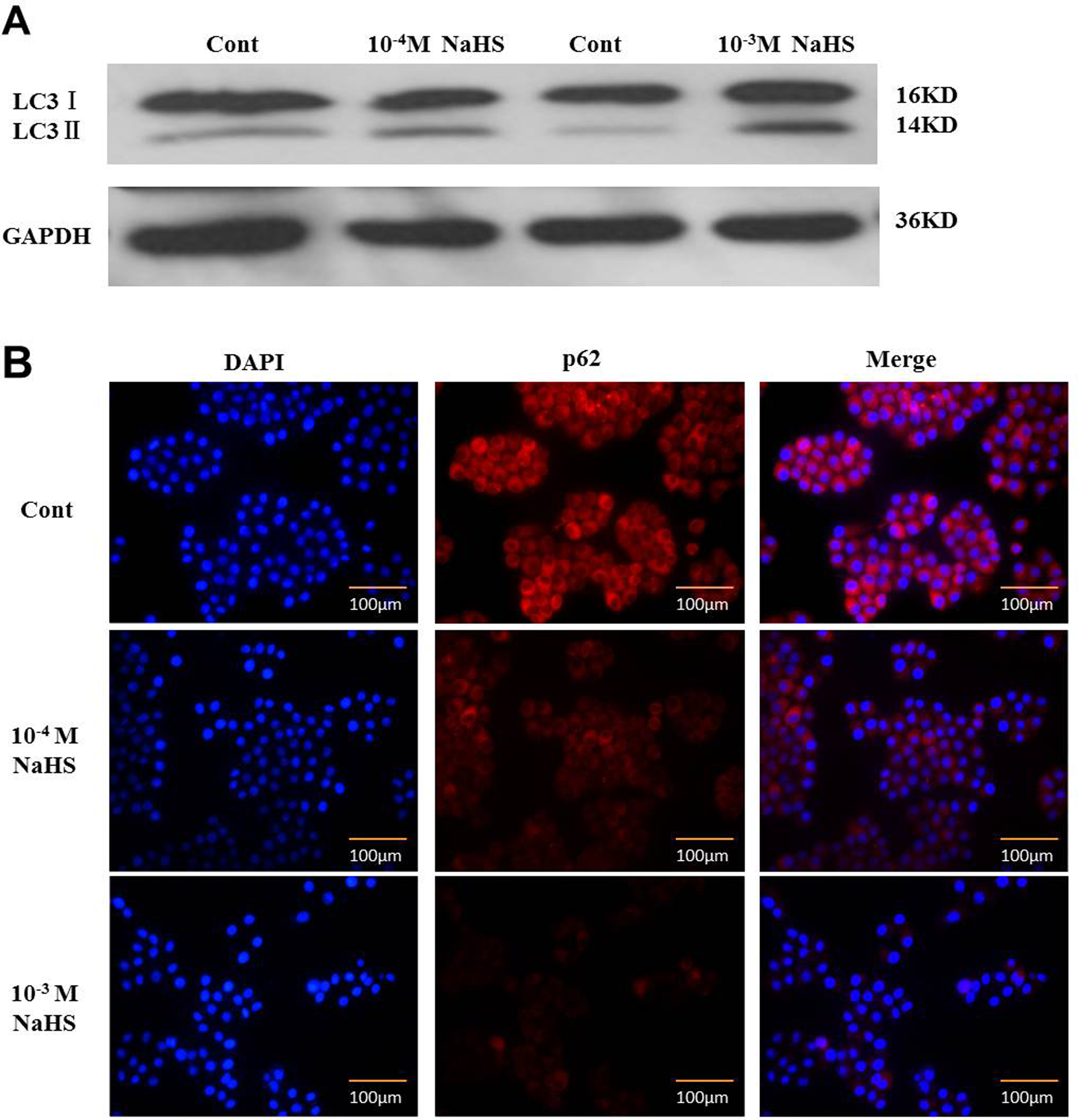

Supplement: Supplementary Figure 1 [file cddis201718x2.tif]
